# Tachycardia and Positive Amphetamine Screen are Associated With True Positive–Methamphetamine Exposures in Pediatric Patients

**DOI:** 10.1016/j.acepjo.2026.100341

**Published:** 2026-02-27

**Authors:** Dana Gans, William King, Lauren Brewer, Michele Nichols, Kathy Monroe, William Rushton

**Affiliations:** 1 Division of Pediatric Emergency Medicine, Department of Pediatrics, University of Alabama Birmingham School of Medicine, Birmingham, Alabama, USA; 2 Office of Medical Toxicology, Department of Emergency Medicine, University of Alabama Birmingham School of Medicine, Birmingham, Alabama, USA

**Keywords:** urine drug screens, pediatrics, drugs of abuse, methamphetamine

## Abstract

**Objectives:**

Methamphetamine exposure is a high-risk condition in pediatric patients. Standard screening often begins with a rapid urine drug screen (UDS) immunoassay, which can yield false-positive results. Confirmatory testing with gas chromatography and mass spectrometry is more accurate, but is not always readily available. This study aimed to identify clinical factors associated with true methamphetamine positivity.

**Methods:**

We conducted a retrospective cohort study at a single children’s hospital, reviewing cases from January 1, 2018, to December 31, 2023. Patients with a positive screening test for methamphetamine, with results from confirmatory testing, were included. Demographic characteristics, laboratory values, urine findings, and clinical presentations were analyzed.

**Results:**

Of 205 patients with a positive UDS, 52 (25%) underwent confirmatory testing. Most were male (n = 28, 54%), and 33 patients (63%) were 0 to 5 years old. Confirmatory testing was positive for methamphetamine in 32 cases (62%). Tachycardia was present in 31 (60%) patients, and 26 (81%) of these were confirmed methamphetamine positive (*P* < .001). Amphetamine copositivity on the UDS was seen in 26 (50%) patients, 25 (96%) of whom were confirmed methamphetamine positive (*P* < .001). Hypokalemia, elevated creatine kinase level, elevated lactate level, and hyperthermia trended toward association, but did not reach statistical significance.

**Conclusion:**

In this cohort, false–positive methamphetamine results on rapid UDSs were common. Tachycardia and concurrent amphetamine positivity were strongly associated with confirmed methamphetamine exposure. Clinicians should be aware of the limitations of immunoassay-based screening and consider confirmatory testing when available.

**The Bottom Line**

Rapid urine drug screening immunoassays are susceptible to false–positive methamphetamine results. This limitation is due primarily to imperfect antibody specificity and cross-reactivity with other substances that share similar chemical structures. Although confirmatory testing is available and more accurate, results are often delayed for several days. During this period, pediatric emergency clinicians may be required to make time-sensitive decisions regarding the validity of screening results, particularly when consideration is being given to notifying child protective services.

In this retrospective analysis, we evaluated clinical variables that were more likely to be associated with true methamphetamine positivity on confirmatory testing. Tachycardia demonstrated a sensitivity of 83.9% and a specificity of 75%. A positive screening urine drug test for amphetamines, a metabolite of methamphetamine, also showed strong diagnostic performance, with a sensitivity of 81.3% and a specificity of 95%.

## 1 Introduction

### 1.1 Importance

Methamphetamine is a dangerous illicit drug that is among the most highly abused substances in the United States.^1^ Adolescent users are at risk for progressing to substance use disorder, with associated criminal involvement and increased morbidity and mortality from medical complications. Children of chronic users are more likely to be neglected and abused, in addition to living in a dangerous environment with exposure to drugs and associated paraphernalia. Infants and toddlers living in homes where methamphetamine is available or produced are particularly at risk due to their smaller size, faster metabolism, prolonged indoor exposure, and propensity to place objects in their mouths.^1^ When children are found to be in a drug-exposed environment, the Department of Human Resources or Child Protective Services typically becomes involved. Particularly in the case of a dangerous illicit drug such as methamphetamine, a child may be removed from the home and placed in kinship or foster care.

Rapid urine drug screens (UDSs) are immunoassay tests that are easy to use and have a quick processing time. Unfortunately, immunoassay UDSs are prone to false–positive methamphetamine results due to poor antibody specificity and cross-reactivity between the antibody and structurally related interfering compounds.^2^^,^^3^ Often referred to as a comprehensive UDS (confirmatory test), gas chromatography/mass spectrometry, high-performance liquid chromatography, or liquid chromatography/tandem mass spectrometry analysis can be ordered to confirm methamphetamine presence in the urine.^4^ Unfortunately, the confirmatory test takes days to weeks to result at many institutions, including our own, potentially delaying intervention in a pediatric patient at risk.

### 1.2 Background

Methamphetamine is primarily metabolized into 2 compounds: inactive hydroxymethamphetamine via aromatic hydroxylation and active amphetamine via demethylation.^4^^,^^5^ In one study of healthy volunteers ingesting methamphetamine, urine remained positive in some cases for up to 7 days postexposure.^6^ In individuals with true methamphetamine exposure, amphetamine, as a major metabolite, is also expected to be detectable on UDSs that report both amphetamine and methamphetamine within hours of exposure.^6^

Methamphetamine increases cytoplasmic catecholamine concentrations by disrupting ion trapping and promoting reverse transport through vesicular membrane transporters.^5^ This leads to elevated synaptic levels of norepinephrine, dopamine, and serotonin, producing the classic sympathomimetic toxidrome.^5^^,^^7^ Clinical features may include hyperthermia, tachycardia, hypertension, dyspnea, mydriasis, diaphoresis, agitation, abnormal motor activity, and psychosis.^1^^,^^7^, ^8^, ^9^ Elevated creatine kinase (CK) and lactate levels may result from neuromuscular activation and vasoconstriction-induced ischemia.^8^ Sympathomimetic substances can also lower serum potassium levels.^10^ Catecholamines induce hypokalemia by stimulating β-adrenergic receptors, which enhance Na+/K+-ATPase activity and promote intracellular potassium shifting.^11^

### 1.3 Goals of This Investigation

This study aimed to identify clinical factors, including vital sign abnormalities, laboratory values, and presentation, that predict true methamphetamine exposure in pediatric patients with a positive UDS. Given that amphetamine is a methamphetamine metabolite, we also assessed whether a positive amphetamine result on the initial UDS increases the likelihood of confirmed methamphetamine on a confirmatory urine drug test.

## 2 Methods

### 2.1 Study Design and Setting

This study is a single-center, retrospective cohort study comparing clinical and laboratory factors of pediatric patients who tested positive for methamphetamine on a rapid UDS. The study location is a high-volume, stand-alone pediatric hospital in the southeastern United States, with an annual pediatric emergency department volume exceeding 70,000. Data were collected over a 6-year–time frame, dating from January 1, 2018, to December 31, 2023. A pediatric emergency physician and a pediatric resident extracted all data. The pediatric emergency physician performed a secondary review of all data to confirm accuracy. Our university’s institutional review board approved this study.

### 2.2 Selection of Participants

Hospital records were obtained for all patients who tested positive for methamphetamine on a UDS and had a confirmatory urine drug test performed during the same emergency department visit or hospital admission. Patients were excluded if confirmatory results were unavailable. The rapid UDS was performed via a qualitative, 1-step–solid-phase immunoassay interpreted via an automated reader (PROFILE-V MEDTOXScan Drugs of Abuse Test System, MEDTOX Diagnostics). The package insert reports that both methamphetamine and amphetamine are reported as positive at concentrations >500 ng/ml.^12^ Confirmatory testing via the comprehensive urine drug test was conducted using liquid chromatography/tandem mass spectrometry (MEDTOX ToxAssure Comprehensive Test, Labcorp). This test detects both methamphetamine and amphetamine at concentrations >50 ng/mL.^13^

### 2.3 Outcomes

The primary outcomes assessed included amphetamine copositivity on immunoassay UDS, vital sign abnormalities, and hypokalemia among patients with a positive methamphetamine result on the initial drug screen. Secondary outcomes included investigating for CK level elevation, hyperlactatemia, and the physician's clinical suspicion for methamphetamine ingestion. We also obtained basic demographic information on age and gender, as well as the date of ingestion. Age was divided into 2 groups of 0 to 5 years and 6 to 19 years to account for patients who would be more likely to have incidental home or exploratory exposure vs patients who may have an intentional exposure.

### 2.4 Measurements

We assigned strict definitions for study variables a priori. Hyperthermia was a temperature ≥ 38°C; tachycardia and severe tachycardia were heart rates above the 90th and 99th percentiles for age, respectively.^14^ Hypertension and severe hypertension were systolic blood pressures above the 90th and 95th percentiles for age, using values for the 50th percentile for height.^15^ Hypokalemia was a serum potassium level < 4.1 mEq/L for infants and < 3.5 mEq/L for children and adolescents. Elevated creatinine kinase level was defined as > 250 U/L, and hyperlactatemia, a serum lactate level > 2 mmol/L. We categorized provider clinical suspicion into 9 groups based on triage chief complaint, documented signs and symptoms, and the concern prompting urine drug screening.

### 2.5 Analysis

We conducted a series of univariate analyses comparing clinical and laboratory values on patients who were UDS methamphetamine positive to identify factors associated with positive methamphetamine on confirmatory drug tests. We stratified vital signs by age group. The Kolmogorov-Smirnov test of normality was used in selecting means or medians for continuous variables of interest. Chi-square analysis (Yates correction where appropriate), Fisher's exact test (with exact probabilities), Mann-Whitney U test (for median age comparison), and t-test of means with Satterthwaite adjustment were the predominant statistical tests used (Epi Info Version 7.2.4.0, CDC). Screening performance metrics were calculated to assess how well either a positive UDS (amphetamine) or a clinical variable (tachycardia) identified a true methamphetamine–positive result. Sensitivity (true-positive rate) and specificity (true-negative rate), along with their respective 95% CIs, were reported. Standard statistical methods were utilized (Microsoft Excel), yielding descriptive statistics (counts and percentages in the setting of categoric data and medians and IQRs for continuous variables) as reported.

## 3 Results

### 3.1 Characteristics of Study Subjects

During the 6-year–study period, 205 patients tested positive for methamphetamine on a rapid UDS. Of those patients, 52 (25%) had a subsequent confirmatory urine drug test obtained and were therefore eligible for the study. Male patients accounted for 28 (54%) of the patients. Thirty-three (63%) patients were in the 0 to 5 years old cohort, and 19 (37%) patients were in the 6 to 19 years old cohort. Twenty-one (40%) patients were < 24 months old, the youngest being 5 months old. There were no statistically significant differences in demographic characteristics between the true–positive and false–positive methamphetamine cohorts.

Of the 52 patients with a methamphetamine-positive screening UDS, 32 (62%) subsequently had a methamphetamine–positive confirmatory test. Thirty-one (60%) of the confirmatory urine drug tests were also positive for amphetamine. Fifteen (47%) of the methamphetamine-positive patients were female, and the median age was found to be 2 (range 0-19) years. Twenty-six (81%) of the confirmed methamphetamine positive patients had a positive result for amphetamine on the screening UDS. Twenty-two (69%) of the confirmed methamphetamine ingestions were in the 0 to 5 years old age group, and 10 (31%) were in the older 6 to 19 years old age group. Demographic information for patients is represented in Table 1.

**Table 1:** Demographic characteristics of pediatric patients whose screening-immunoassay UDS returned positive for methamphetamine.

Characteristic	Confirmatory methamphetamine negative n = 20 (38%)	Confirmatory methamphetamine positive n = 32 (62%)	Total n = 52 (100%)	*P* value
Gender (male, female)	Female = 9 (45%)	Female = 15 (47%)	Female = 24 (46%)	χ^2^ = 0.00, *P* = 1.00
Median age, y (range)	4 (1-19)	2 (0-19)	2 (0-19)	H = 2.0, *P* = .15
Age, 0-5 y	11 (55%)	22 (69%)	33 (63%)	χ^2^ = 0.49, *P* = .48
Age, 6-19 y	9 (45%)	10 (31%)	19 (37%)	

UDS, urine drug screen.

### 3.2 Main Results

Across all age groups, 31 of 52 subjects had heart rates exceeding 90% of the age-adjusted standard, the majority of which (n = 26, 81%) were associated with true methamphetamine positivity (χ^2^ =13.9, *P* < .001, using Yates’ continuity correction). When analyzed by age cohorts, this association remained statistically significant in both the 0 to 5 year group (*P* = .006) and the 6 to 19 year group (*P* = .02). Tachycardia demonstrated a sensitivity of 83.9% (95% CI, 66.3%-94.6%]) and a specificity of 75% (95% CI, 50.0%-91.3%). These findings are summarized in Table 2.

**Table 2:** Variables of pediatric patients whose screening-immunoassay UDS returned positive for methamphetamine.

Variable	Confirmatory methamphetamine negative n = 20	Confirmatory methamphetamine positive n = 32	Total n = 52	*P* value
Heart rate				
All ages, y				
>90th % for age	5 (25%)	26 (81%)	31 (60%)	χ^2^ = 13.9, *P* = .0002
>99th % for age	2 (10%)	18 (56%)	20 (38%)	0.001
Age 0-5 y				
>90th% for age	4 (36%)	19 (86%)	23 (70%)	0.006
>99th% for age	2 (18%)	14 (64%)	16 (48%)	0.026
Age 6-19 y				
>90th % for age	1 (11%)	7 (70%)	8 (42%)	0.020
>99th % for age	1 (11%)	4 (40%)	5 (26%)	0.030
Hyperthermia	1 (5%) +	5 (16%)	6 (12%) +	0.384

	Confirmatory methamphetamine negative n = 19	Confirmatory methamphetamine positive: n = 32	Total n = 51	*P* value
Blood pressure				
All ages				
>90th % for age	16 (84%) +	25 (78%)	41 (80%) +	0.725
>95th % for age	13 (68%) +	23 (72%)	36 (71%) +	1.000
Age 0-5 y				
>90th % for age	10 (91%)	20 (91%)	30 (91%)	1.000
>95th % for age	9 (82%)	19 (86%)	28 (85%)	1.000
Age 6-19 ys				
>90th % for age	6 (75%) +	5 (50%)	11 (58%) +	0.367
>95^th^ % for age	4 (50%) +	4 (40%)	8 (42%) +	1.000

	Confirmatory methamphetamine negative n = 15	Confirmatory methamphetamine positive n = 30	Total n = 45	*P* value
Hypokalemia	1 (7%) +	9 (30%) +	10 (22%) +	0.132

	Confirmatory methamphetamine negative n = 7	Confirmatory methamphetamine positive n = 13	Total n = 20	*P* value
Hyperlactatemia	1 (14%) +	7 (54%) +	8 (40%) +	0.157

	Confirmatory methamphetamine negative n = 2	Confirmatory methamphetamine positive n = 15	Total n = 17	*P* value
Elevated creatinine kinase level	1 (50%) +	14 (93%) +	15 (88%) +	0.228

UDS, urine drug screen.

See methods for definitions of tachycardia, hypertension, hyperthermia, hyperlactatemia, and creatinine kinase level elevation. *N* denotes the number of cases where the clinical variable was able to be obtained from the chart. + indicates that not all cases reported the variable. Tachycardia demonstrated a sensitivity of 83.9% (95% CI, 66.3%-94.6%) and a specificity of 75% (95% CI, 50.0%-91.3%).

Analysis of additional clinical variables further reinforced the association between tachycardia and true positive–methamphetamine results. In the 0 to 5 year cohort, the mean heart rate was 132 beats per minute (bpm) among patients with a negative methamphetamine confirmatory screen and 166 bpm among those with a positive methamphetamine confirmatory screen, resulting in a difference of 34 bpm (95% CI, 11.3-56.0). In the 6 to 19 year cohort, the mean heart rate was 84 bpm for methamphetamine-negative patients and 104 bpm for methamphetamine-positive patients, though this 20 bpm difference did not reach statistical significance (95% CI, −6.8 to 46.8).

Of the 51 patients with recorded blood pressures, 41 (80%) demonstrated hypertension during evaluation. Similar rates of elevated blood pressure were observed between the confirmed methamphetamine-positive and methamphetamine-negative groups, even after adjusting for age (Table 2). Body temperature was recorded in 50 patients, and hyperthermia was identified in 6 cases. Of the 6 reported cases of hyperthermia, 5 occurred in patients within the confirmed methamphetamine-positive group, compared with 1 in the methamphetamine-negative group (*P* = .384).

Potassium serum concentrations were reported for 45 patients, of whom 10 were hypokalemic. Among patients with a confirmed positive methamphetamine drug test, 9 (30%) were hypokalemic, compared with 1 patient (7%) in the methamphetamine-negative group (*P* = .132). Lactate concentrations were available for 20 patients; 7 of the confirmed methamphetamine-positive patients (54%) had lactate levels > 2 mmol/L, whereas only 1 patient (14%) in the methamphetamine-negative group demonstrated an elevated lactate level (*P* = .157). CK levels were collected for 17 patients, with 15 showing levels above 250 IU/L. Although nearly all of the 15 patients with elevated CK levels had confirmed methamphetamine exposure (n = 14), this association also did not reach statistical significance (*P* = .228).

Of the 32 patients in this cohort who had methamphetamine confirmed positive on the comprehensive test, 26 patients (81%) also screened positive for amphetamines on the initial rapid immunoassay (Table 3). Of these patients, 25 (96%) were in the confirmed methamphetamine-positive group, whereas only 1 (4%) was confirmed in the amphetamine-negative group (*P* < .001). Amphetamine positivity on the screening UDS also demonstrated a sensitivity of 81.3% (95% CI, 63.4%-92.8%) and a specificity of 95% (95% CI, 75.1%-99.9%). The methamphetamine–positive confirmatory drug tests were reviewed to assess for amphetamine copositivity to further determine whether the amphetamine-positive results on the drug screen were true positive vs a false positive resulting from cross-reactivity with methamphetamine itself in the immunoassay. Of the 32 patients that had confirmed methamphetamine in their sample, 31 (97%) also had confirmed amphetamine, suggesting that amphetamine positivity on the UDS was not due to methamphetamine cross-reactivity, but a true positive–amphetamine result.

**Table 3:** Amphetamine urine drug screens.

Amphetamine result	Confirmatory methamphetamine positive (n = 32)	Confirmatory methamphetamine negative (n = 20)
Immunoassay urine screen		
Positive	26 (81%)^a^	1 (5%)^a^
Negative	6 (19%)^a^	19 (95%)^a^

Amphetamine positivity on the screening urine drug screens demonstrated a sensitivity of 81.3% (95% CI, 63.4%-92.8%) and a specificity of 95% (95% CI, 75.1%-99.9%).

a = *P* < .001.

**Figure 1:**
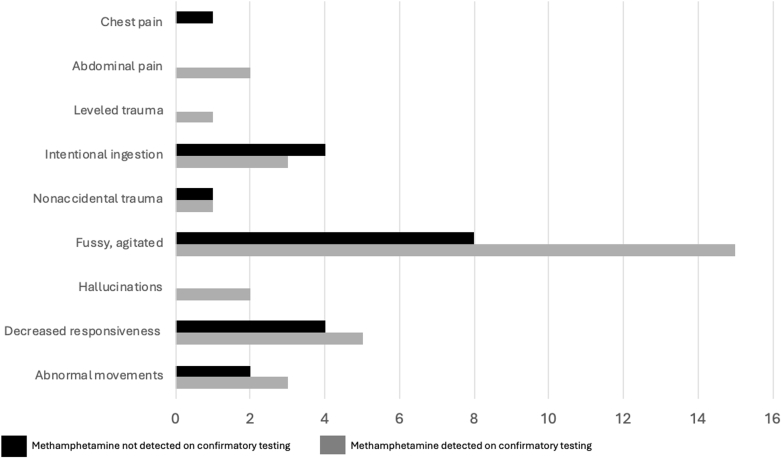
History and physical examination findings reported by the emergency department or admission documentation on pediatric patients who screened positive for methamphetamine. Dark bars indicate negative-methamphetamine results on the confirmatory test, whereas gray bars represent cases in which methamphetamine was detected. No findings were statistically significant.

Provider suspicion for ingestion of a substance, whether specifically methamphetamine or not, was assessed by combining the patient’s presenting signs and symptoms with their chief complaint. Patients were then placed into 1 of 9 categories, as seen in Figure 1 (chest pain, abdominal pain, level of trauma, intentional ingestion, nonaccidental trauma, fussy/agitated, hallucinations, decreased responsiveness, and abnormal movements). Of the 23 patients described as either fussy or agitated, 15 (65%) were confirmed positive for methamphetamine, but this did not reach significance (*P* = .4).

**Figure 2:**
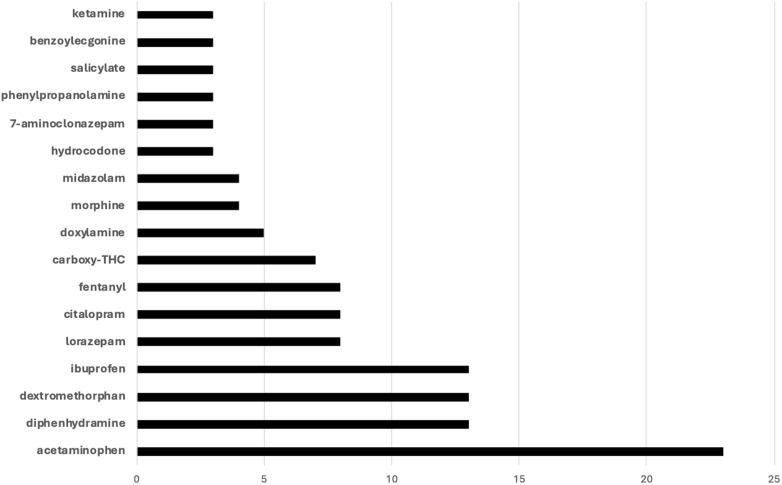
Frequency of other xenobiotics on confirmatory testing. Mirtazapine, metoprolol, ephedrine/pseudoephedrine, guaifenesin, bupropion, alpha-hydroxymidazolam, naproxen, hydroxyzine, aripiprazole, lidocaine, and methylphenidate were each detected in 2 samples. Other substances, including lamotrigine, brompheniramine, tapentadol, fluoxetine, gabapentin, levetiracetam, tramadol, caffeine, desalkylflurazepam, and dihydrocodeine, were each detected once.

**Figure 3:**
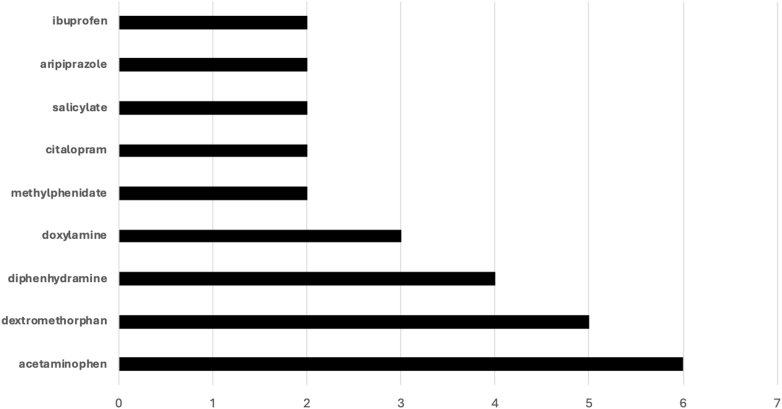
Frequency of other xenobiotics when there was no methamphetamine detected on the confirmatory test. Other substances, including lidocaine, ketamine, benzoylecgonine, carboxy-THC, lamotrigine, metoprolol, lorazepam, brompheniramine, dihydrocodeine, naproxen, 7-amino clonazepam, hydroxyzine, and hydrocodone, were each detected once. THC, tetrahydrocannabinol.

On the confirmatory screen, 38 xenobiotics, in addition to methamphetamine and amphetamine, were detected. The top 5 most common substances were acetaminophen (n = 23), dextromethorphan (n = 13), diphenhydramine (n = 13), ibuprofen (n = 13), and fentanyl (n = 8) (Fig 2). When specifically looking at the cohort where methamphetamine was not detected on the confirmatory screen, the most common other xenobiotics encountered were acetaminophen (n = 6), dextromethorphan (n = 5), diphenhydramine (n = 4), doxylamine (n = 3), and salicylates (n = 2) (Fig 3).

## 4 Limitations

This study has several limitations. It was conducted at a single institution, although a major children’s hospital in a state with 5 million residents and a high prevalence of methamphetamine use.^16^ Many patients were missing multiple laboratory values, and some did not have a complete set of vital signs. Medical decision making and descriptions of patients’ symptoms were also inconsistent, limiting the ability to make stronger associations with patient complaints and true methamphetamine-positive results. In addition, only 52 (25%) of the 205 patients who were positive for methamphetamine on rapid UDSs were referred for confirmatory testing, introducing potential nonrandom referral bias and limiting the representativeness of the confirmed cohort. Further bias was likely introduced by the lack of standardization in ordering UDSs. Clinicians obtained them based on individual gestalt for a wide range of reasons, including altered mental status, vital sign abnormalities, suspected ingestions, and neurologic symptoms, among others. Finally, the limited number of inclusion cases reduced the statistical power of the study.

## 5 Discussion

Our study identified clinical correlates that may be useful in distinguishing a true methamphetamine ingestion when an immunoassay UDS result is positive. Heart rates exceeding both the 90^th^ percentile and 99^th^ percentile age-adjusted standards were associated with true positive–methamphetamine exposure confirmed on comprehensive urine drug testing. This association was statistically significant in both pediatric age groups. Quantitatively, children aged 0 to 5 years with confirmed methamphetamine exposure had average heart rates that were 30 bpm higher than those with false-positive results. Among older children and adolescents, those with methamphetamine exposure also had higher average heart rates by about 20 bpm, though this difference did not reach statistical significance. High-quality prior reports examining the association between tachycardia and true methamphetamine exposure in the pediatric population are lacking. In a retrospective analysis of trauma patients undergoing operative treatment, true methamphetamine usage was not associated with tachycardia. This analysis did have similar findings showing no association with methamphetamine usage and hypertension. However, multiple differences exist between this report and ours: adult patient population, predominantly trauma patients, and no confirmatory testing on methamphetamine immunoassays.^17^

A positive amphetamine result on the rapid immunoassay was strongly associated with a true methamphetamine finding on confirmatory testing, demonstrating both a sensitivity of 81.3% and a specificity of 95%. This relationship is expected, as amphetamine is a known metabolite of methamphetamine. Although most methamphetamine is excreted unchanged, approximately 4% to 7% of methamphetamine is excreted in the urine as D-amphetamine.^6^ When both the parent compound and its metabolite are detected on the initial UDS, the likelihood of a true-positive result increases. A previous study of healthy adults also showed that amphetamine may be detected in under an hour following methamphetamine ingestion.^6^ Furthermore, given methamphetamine’s half-life, amphetamine may remain positive for several days. Interestingly, amphetamine was identified more frequently on confirmatory testing than on initial immunoassays, likely due to the confirmatory assay’s lower detection threshold compared with the higher cutoff values of immunoassays. Clinically, this suggests that a rapid UDS positive for methamphetamine alone, without amphetamine, may indicate either a very early ingestion, a potential false positive, or an amphetamine concentration below the cutoff.

Sympathomimetic signs and symptoms are quite helpful when addressing the chief complaint and assessing the patient, including neuropsychiatric complaints. Fussy and agitated behavior should be concerning in toddlers, as they have the tendency to place small objects in their mouths and could therefore unintentionally ingest the toxin while being too poorly verbal to state what happened. Many teenagers present with aggressive behavior, and a drug screen may be considered to assess for possible substance use.^18^

Hypokalemia has been reported in several case reports from phenylethylamine and methamphetamine use, resulting from intracellular shift and retention of potassium from beta-adrenergic activation. This reaction to adrenergic surge is limited to the half-life of methamphetamine and could be missed in late-presenting exposures.^19^^,^^20^ It is plausible that hypokalemia contributes to muscle weakness and altered mental status, which may correspond to the decreased responsiveness observed in some of our patients.^21^ However, because potassium levels were obtained in only a small subset of patients, this association cannot be firmly established in our study. Notably, though, hypokalemia was more common among patients with a positive confirmatory methamphetamine test than among those with negative screens. Hyperthermia, elevated CK levels, and elevated lactate levels also showed trends toward an association with true methamphetamine positivity, though these did not reach statistical significance due to the limited power of the study.

Often, social services, Child Protective Services, or the Department of Human Resources are consulted and involved in evaluating whether a child is ultimately safe to be discharged home with the parents in the setting of an exposure. One of the most concerning results of our study is the high rate (38%) of false–positive methamphetamine results that were reported in our analysis. Consequently, it behooves the clinician to understand and convey the limitations of the rapid UDS, particularly the potential for false–positive methamphetamine results. When screening urine drug findings do not align with the patient’s clinical presentation or history, or when there is any concern about the validity of the results, confirmatory testing should be pursued. Additionally, as noted in the introduction, young children are at increased risk, and clinicians should maintain a low threshold for obtaining confirmatory testing in this population.

In summary, this retrospective pediatric study conducted at a single institution demonstrated that tachycardia and amphetamine positivity on screening rapid immunoassays were associated with confirmed methamphetamine exposure. Although hyperthermia, elevated lactate levels, hypokalemia, and increased CK levels were more common in the confirmed methamphetamine cohort, these differences were not statistically significant. It is essential to acknowledge the limitations and potential false-positive rates inherent in screening rapid immunoassays when interpreting these laboratory findings.

## Funding and Support

By *JACEP Open* policy, all authors are required to disclose any and all commercial, financial, and other relationships in any way related to the subject of this article as per conflict of interest guidelines (see www.icmje.org). The authors have stated that no such relationships exist.

## Conflict of Interest

All authors have affirmed they have no conflicts of interest to declare.
